# Developing a peptide to disrupt cohesin head domain interactions

**DOI:** 10.1016/j.isci.2023.107498

**Published:** 2023-07-28

**Authors:** Maria Elias, Samar Gani, Yana Lerner, Katreen Yamin, Chen Tor, Adarsh Patel, Avi Matityahu, Moshe Dessau, Nir Qvit, Itay Onn

**Affiliations:** 1 Chromosome Instability and Dynamics Lab, Azrieli Faculty of Medicine, Bar-Ilan University, Safed, Israel; 2 Protein-Protein Interactions Lab, Azrieli Faculty of Medicine, Bar-Ilan University, Safed, Israel; 3 The Lab for Structural Biology of Infectious Diseases, Azrieli Faculty of Medicine, Bar-Ilan University, Safed, Israel

**Keywords:** Protein, Molecular biology, Cell biology

## Abstract

Cohesin mediates the 3-D structure of chromatin and is involved in maintaining genome stability and function. The cohesin core comprises Smc1 and Smc3, elongated-shaped proteins that dimerize through globular domains at their edges, called head and hinge. ATP binding to the Smc heads induces their dimerization and the formation of two active sites, while ATP hydrolysis results in head disengagement. This ATPase cycle is essential for driving cohesin activity. We report on the development of the first cohesin-inhibiting peptide (CIP). The CIP binds Smc3 *in vitro* and inhibits the ATPase activity of the holocomplex. Treating yeast cells with the CIP prevents cohesin’s tethering activity and, interestingly, leads to the accumulation of cohesin on chromatin. CIP3 also affects cohesin activity in human cells. Altogether, we demonstrate the power of peptides to inhibit cohesin in cells and discuss the potential application of CIPs as a therapeutic approach.

## Highlights

• Cohesin inhibiting peptide (CIP) binds Smc3 and inhibits ATPase activity *in vitro*
• Treating yeast cells with CIP results in premature sister chromatid cohesion
• CIP induces cohesin accumulation on chromatin in yeast
• In human cells, CIP increases mitosis time

## Graphical abstract

**Figure:**
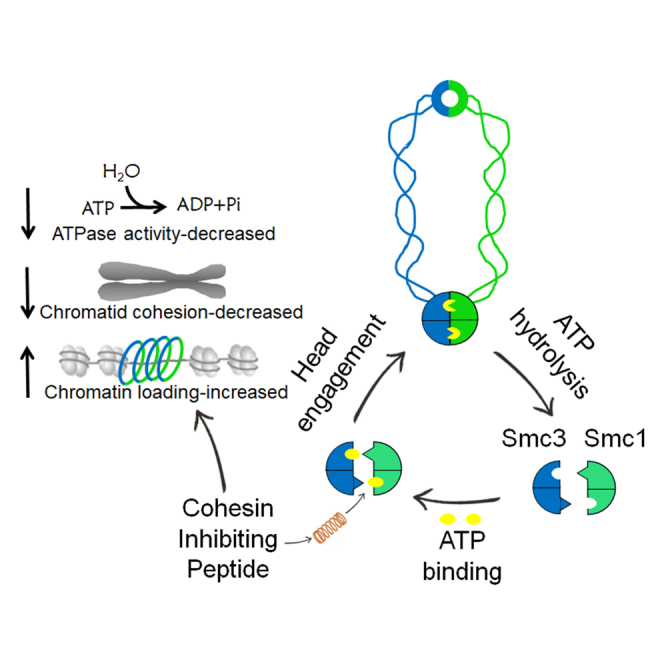

## Introduction

The three-dimensional organization of chromatin is important for maintaining genome stability and dynamics.^1^ Cohesin, evolutionarily conserved structural maintenance of chromosome (SMC) complex, plays key functions in organizing higher-order chromatin structures. Cohesin extrudes interphase chromatin into loops and other higher-order structures that play roles in the initiation of DNA replication, the regulation of gene expression, and DNA repair. After DNA replication, cohesin tethers the replication products, known as sister chromatids, to ensure the fidelity of their segregation into daughter cells during mitosis.^2^^,^^3^^,^^4^

**Figure 1:**
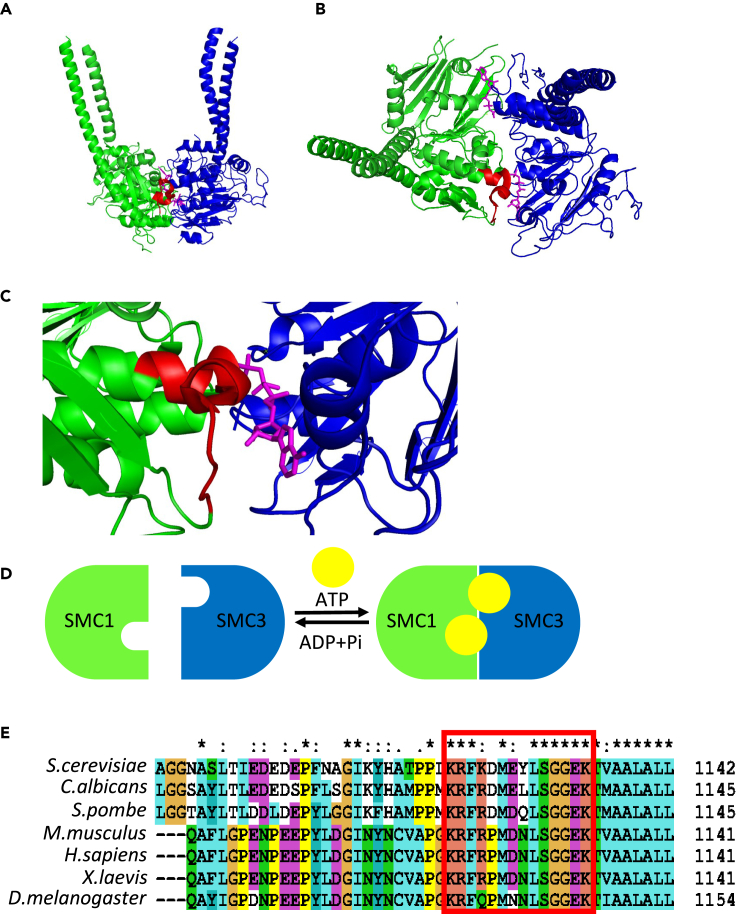
Cohesin’s SMC head domain The atomic structure of Smc1 (green) and Smc3 (blue). ATP molecules are in magenta (PDB: 6YUF). The CIP3-related region in Smc1 is in red. (A) Side view. (B) Top view. (C) Zoom-in into the CIP3-related region. (D) Schematic of the head engagement- and disengagement-inducing ATPase cycle. (E) Protein sequence alignments (ClustalX) of Smc1 show the conservation of the regions corresponding to CIP3.

Cohesin is a four-subunit, ring-shaped complex composed of Smc1, Smc3, a kleisin (in yeast, Mcd1/Scc1; in mammalians, RAD21), and Scc3 (STAG in mammalians).^5^ The first two subunits, the SMC proteins, adopt an elongated structure composed of two globular domains connected by an extended coiled-coil region. The structure is formed by a foldback of the polypeptide on itself. The foldback region creates the first globular domain, known as the hinge. The second globular domain called the head, is formed on the opposing side of the protein by the adjacent amino- and carboxy-termini (Figures 1A–1C). The head harbors two-halves of nucleotide-binding domains (NBD).^6^^,^^7^ An extended coiled-coil region with occasional breaks separates the hinge and head domains. The holocomplex is assembled by dimerization of Smc1 and Smc3 through their hinges. The kleisin forms a bridge between the Smc1 and Smc3 heads, restricting their free movement and closing the tripartite core. The fourth subunit, Scc3, interacts with the kleisin, which also serves as an interaction hub for cohesin regulatory factors Scc2/Scc4, Wpl1, and Pds5.^8^

Cohesin NBD is of the ATP binding cassette (ABC)-type composed of three highly conserved motifs.^9^ Walker A and Walker B mediate nucleotide binding, and a Signature motif is involved in ATP to ADP hydrolysis. In cohesin, two active ATPase domains are assembled by bringing together the Walker A motif from one SMC protein together with the Walker B and Signature motifs located in the opposing SMC protein. DNA binding to cohesin induces the physical engagement of the Smc1 and Smc3 heads and stimulates ATP hydrolysis, which is followed by head disengagement (Figure 1D).^10^^,^^11^^,^^12^^,^^13^^,^^14^^,^^15^^,^^16^^,^^17^ The ATPase cycle is associated with conformational changes in cohesin that control the compartmentalization of the structure.^16^^,^^18^^,^^19^ Genetic manipulation of ATPase sites and biochemical studies conducted with non-hydrolyzed ATP analogs have revealed that cohesin’s ATPase activity is essential for cohesin loading onto chromatin, loop extrusion, and sister chromatid cohesion.^10^^,^^11^^,^^13^^,^^16^^,^^20^ ATP hydrolysis is also important for cohesin dissociation from chromatin.^21^

The ATPase activity of cohesin is suppressed by the acetylation of two adjacent lysines located in the head domain of Smc3 by Eco1 acetyltransferase, occurring during the S-phase of the cell cycle, this modification inhibits the ATPase cycle and shifts cohesin from its unstable DNA-binding form to a stable binding mode, enabling sister chromatid tethering.^2^^,^^22^^,^^23^^,^^24^^,^^25^ The ATPase activity of cohesin and its role in the mechanism of action has been the focus of several studies. However, little attention has been paid to the importance of the interactions between the Smc1 and Smc3 heads.

Using peptides to inhibit protein-protein interactions (PPIs) is an emerging research field presenting exciting opportunities for new therapeutic approaches. Peptides derived from the interaction site can bind to the protein surface with high affinity, creating a protein/peptide complex, which disrupts the binding interface between the two interacting proteins. This results in the inhibition of the construction of an active dimer. In a cellular environment, successful competition of a PPI-inhibiting peptide with the native binding partner on an interaction domain has two prerequisites. First, the native PPI should be dynamic, allowing exposure of the binding surface and the opportunity for the peptide to compete on it with the native partner. Second, once the peptide binds to its target, the binding affinity should be high to prevent its deposition from the interaction site.^26^^,^^27^^,^^28^

Our aim was to develop a peptide to inhibit cohesin activity. The cohesin SMC complex’s head domain interactions fulfill the first condition. Therefore, the remaining challenge was to design a peptide that competes with the native partners and prevents head engagement. We introduce the first yeast cohesin head domain-inhibiting peptide. Its sequence is derived from a conserved region in Smc1 (Figure 1E). The peptide binds to Smc3 and inhibits cohesin functions in cells. Notably, inhibiting Smc1/Smc3 head engagement results in the accumulation of cohesin on chromosomes. Treating human cells with the yeast-derived peptide revealed that it might also impede cohesin in these cells. This work demonstrates the potential of peptides to inhibit cohesin *in vitro* and in cells and provides insight into the molecular basis by which cohesin tethers chromatin.

## Results

### Peptide design and screening for peptide-inhibiting activity in cells

We designed three cohesin-inhibiting peptides (CIPs, Table 1) to inhibit Smc1/Smc3 protein-protein interaction. CIP1 and CIP2 were derived from conserved regions in Smc3, while CIP3 was derived from a conserved region in Smc1 (Figure 1E). To explore the biological effect of the peptides on cohesin, we sought to overexpress them in cells. For that purpose, we constructed a centromeric plasmid—pME4 (CIP1), pME2 (CIP2), and pME3 (CIP3), in which the peptide coding sequence was inserted after an ATG codon and under the control of a GAL promoter. Cells were transformed with the plasmids and grown in an SC-URA medium. In addition, we transformed cells with a control plasmid (pIO014).

**Table 1:** Peptides list

Name	Peptide sequence	Derived from	Target
CIP1	MKQNEQLHVEQLSGGQKTV	Smc3, 1116-1133	Smc1
CIP2	MIIGSNGSGKSN	Smc3, 30-40	Smc1
CIP3	MKRFKDMEYLSGGEKT	Smc1, 1121-1135	Smc3
CIP3-TAT	KRFKDMEYLSGGEKT-GGYGRKKRRQRRR	Smc1, 1121-1135	Smc3
ContP	FITC^a^-SGYGRKKRRQRRR-GGGLNPYWMETFT	N/A	N/A

a Fluorescein isothiocyanate.

**Figure 2:**
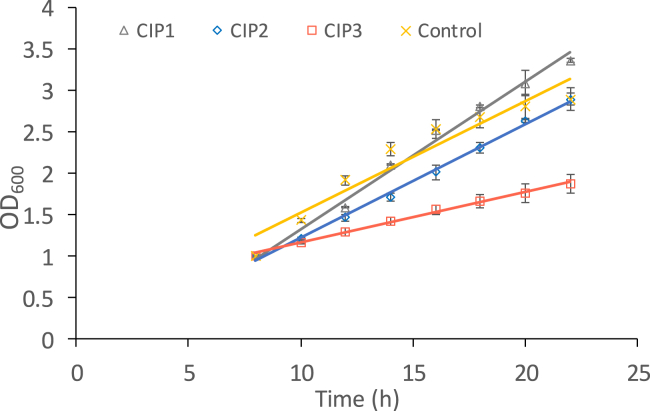
CIP3 causes cell growth delay Strains yIO1000 (control) and yME-031 (pGAL-CIP1), yME-016 (pGAL-CIP2), and yME-019 (pGAL-CIP3) cells were grown in a galactose-containing medium to induce peptide expression. Cell growth was monitored every 120 min to measure the optical density of the culture at 600 nm. The growth rate in the logarithmic growth phase was calculated. The results of a representative experiment are shown. Multiple linear regression revealed no significant decrease in growth rate for cells expressing CIP1 (B = 0.015, SE = 0.119, p = 0.898) or CIP2 (B = −0.290, SE = 0.119, p = 0.022) in comparison with the control. A significant decrease in growth rate was found in cells expressing CIP3 (B = −0.727, SE = 0.119, p < 0.001).

Strains yIO1000 (control) and yME-031 (pGAL-CIP1), yME-016 (pGAL-CIP2), and yME-019 (pGAL-CIP3) cells were diluted in SC-URA galactose medium, and their growth rate was measured by optical density at 600 nm every 120 min. Cohesin is essential for cell division, so we expected that inhibition of cohesin activity by expressing a CIP would affect the cell growth rate. We found a similar logarithmic growth rate of cell cultures with the control plasmid, CIP1, or CIP2. However, cells overexpressing CIP3 showed a weak growth delay compared to the cells carrying the control plasmid, CIP1, or CIP2, indicating that expression of CIP3 may disturb cohesin activity (Figure 2).

The weak effect of CIP3 expressed in cells may relate to several reasons, including low expression levels, low translation efficiency, or cellular instability of short mRNAs and peptides. We tried to increase cellular mRNA stability by using a plasmid containing the T2A ribosome-skipping sequence under GAL promoter control.^29^^,^^30^^,^^31^ Two genes cloned in tandem and separated by a T2A linker were expressed as a single mRNA. The ribosome skips the T2A sequence during translation, resulting in two separate proteins (Figure S1A. Peptide expression from a plasmid containing T2A ribosome-skipping sequence, related to Figure 2). To test the system, we cloned green fluorescent protein (GFP) and mCherry in tandem, separated by the T2A sequence. Cells carrying the plasmids were grown in SC-URA with either noninducing raffinose or inducing galactose. No fluorescence was detected in cells grown in raffinose, while green and red emissions were observed in those grown in galactose (Figure S1B. Peptide expression from a plasmid containing T2A ribosome-skipping sequence, related to Figure 2). Both GFP and mCherry proteins were detected by western blot analysis of extracts from these cells (Figure S1C. Peptide expression from a plasmid containing T2A ribosome-skipping sequence, related to Figure 2).

We inserted CIP3 followed by GFP into the T2A vector and validated mRNA expression by qPCR using a forward primer corresponding to CIP3 and a reverse primer in the GFP and protein expression by detecting green emissions from the cells (Figures S1D and S1E. Peptide expression from a plasmid containing T2A ribosome-skipping sequence, related to Figure 2). Next, we tested the effect of CIP3 expression on cell growth, as aforementioned. However, the inhibition fold was not improved compared to the previous experiment (Figure 2), suggesting that short mRNA degradation is not the reason for the weak peptide inhibition activity in cells. Increasing the efficiency of cohesin inhibition by using this approach remains a future challenge.

### CIP3 binds to Smc3 *in vitro*

**Figure 3:**
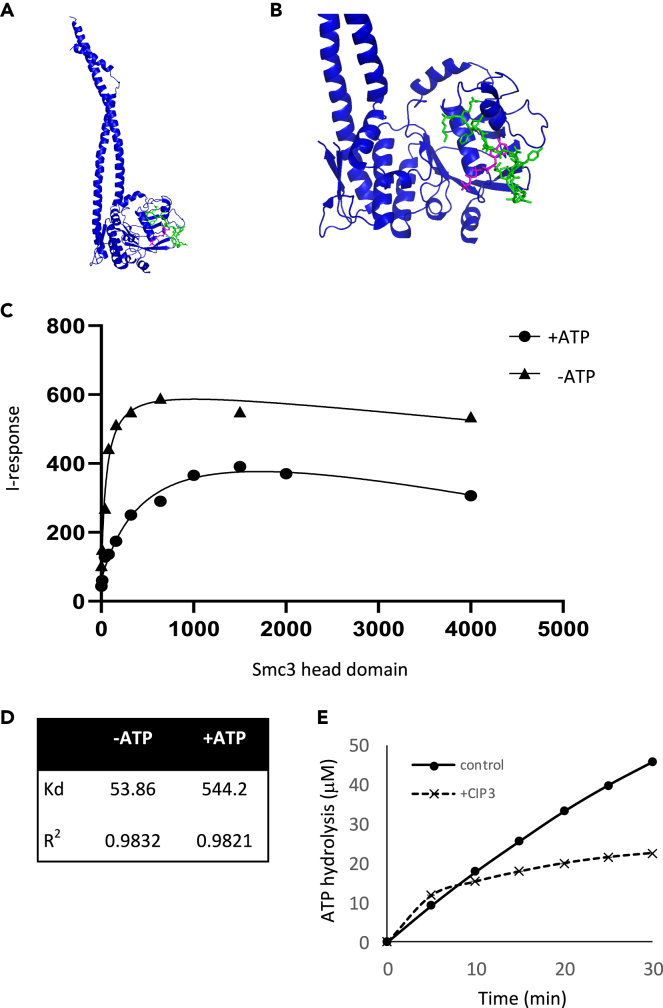
CIP3-TAT binds to Smc3 *in vitro* (A) A structural model generated by HPEPDOC 2.0 server of CIP3-Smc3 head domain docking. Smc3 is in blue, ATP is in magenta, and CIP3 is in green. (B) Zoom in into the Smc3-CIP3 docking region. (C) The kinetic binding of the peptide, CIP3-TAT, to Smc3 head domain protein was explored by FEB Agile R100. 500 nM peptide was immobilized on the sensor chip, and the analyte, Smc3 head domain, in various concentrations (0.02–4 mM) was applied in solution to the chip. A graph showing the I-response in each analyte concentration without ATP (triangles) and with ATP (10 mM, circles). (D) K_d_ (in nM) and R^2^ values were calculated from the results shown in A. (E) Time course analysis of ATP. Hydrolysis by cohesin with or without CIP3.

We decided to focus our efforts on CIP3, as preliminary analysis suggested that CIP1 and CIP2 do not have a detectable biological effect. The CIP3 sequence is derived from the C-terminal region of Smc1. The 15 amino acid sequence is conserved, encoding part of a short loop and the beginning of an alpha helix located at the bottom of the head domain (Figures 1A–1C). It has been suggested that this region is involved in the interaction with Smc3.^12^^,^^17^ We used the HPepDock 2.0 server^32^^,^^33^^,^^34^^,^^35^ to predict the docking site of CIP3 on Smc3. The coordinates of the Smc3 structure (PDB: 4ux3)^36^ were entered as the receptor input and CIP3 as the sequence as the peptide input and the analysis was done using the default parameter. The top model with a docking score of -172.174 shows the peptide stretches in the Smc3 interaction interface, blocking the ATP binding site (Figures 3A and 3B). Two models emerge from this result. CIP3 may interfere with ATP binding to the Walker A site in Smc3. Alternatively, it may misplace the hydrolysis motif in Smc1 and inhibit that ATPase activity. It is important to note that dynamics models are required to understand the structural basis of the Smc3-CIP3 complex fully, as well as experimental validation of the model.

Accordingly, we explored the ability of CIP3 to bind Smc3 *in-vitro*. We synthesized CIP3-TAT and purified it (Figures S2A and S2B. Purification of CIP3-TAT and Smc3 head domain, related to Figure 3), and the kinetic binding of the peptide to a purified Smc3 head domain (Figures S2C and S2D. Purification of CIP3-TAT and Smc3 head domain, related to Figure 3) was explored by FEB Agile R100 (Figures 3C and 3D). The binding kinetics is determined by measuring the current of the chip that is changed when the analyzed molecules form a complex. The peptide was immobilized on the sensor chip, and the analyte, purified Smc3 head domain, was applied in solution to the chip. The changes in the current were monitored as increasing concentrations of analyte were applied to the chip. The dissociation constant (K_d_) of Smc3/CIP3 was 53.86 ± 0.03 nM. ATP binding reduces affinity and may allow the Smc1 head to displace CIP3 from the interaction site. Importantly, ATP was not hydrolyzed, as the active ATPase domain assembly requires Smc1, but ATP binding to Smc3 *in vitro* induces a conformational change in the protein.^19^ Therefore, we sought to repeat the binding experiment in the presence of ATP. The effect of ATP on CIP3 binding was explored by incubating Smc3 with ATP before measuring the Kd. The Kd of CIP3-TAT binding to Smc3 in the presence of ATP increased about 10-fold to 544.20 ± 0.06 nM compared to the Kd of the non-ATP-bound protein (53.86 ± 0.03 nM). This difference in affinities indicates that CIP3 binding to the ATP-free form of Smc3 is preferred.

We validated the *in vitro* binding of CIP3 to Smc3 by exploring the formation of the complex in a yeast cell extract in which Smc3 was fused to GFP. Protein extract from the yAM-945 strain containing Smc3-GFP was supplemented with 10 μM CIP3-TAT and analyzed by microscale thermophoresis (MST) in binding mode. In this assay, the migration of a fluorescent protein in a microscale temperature gradient is monitored. Changes in the physical-chemical properties of the protein, induced by interaction with another protein or a small molecule, will change the migration properties of the protein. While biochemical constants of the binding cannot be determined under these conditions, as the concentration of Smc3-GFP in the extract is undetermined. However, the result of this analysis supported the formation of the Smc3/CIP3 complex (Figure S3. Binding of CIP3-TAT to cohesin by microscale thermophoresis, related to Figure 3). Thus, the assay showed that CIP3 binds cohesin holocomplex in a crowded molecular environment.

### CIP3 inhibits cohesin ATPase activity

The formation of the Smc3-CIP3 complex likely affects the ATPase activity. To explore this possibility, we expressed and purified *S. cerevisiae* cohesin holocomplex and the loader from yeast cells^37^ (Figure S4. Purification of cohesin holocomplex and loader, related to Figure 3), and we measured the ATPase rate of cohesin in the presence of cohesin, the loader DNA, and ATP, as previously described.^37^ Cohesin was preincubated without or with CIP3, and the accumulation of free Pi was measured over 30 min. In the absence of CIP3, Pi accumulation was constant over time, indicating continuous ATP hydrolysis. Pi accumulation in the presence of CIP3 was similar to the control. However, after 5 min, Pi accumulation dropped sharply, indicating that ATPase activity was inhibited. The delay in the inhibition suggests that head disengagement is required to allow the peptide to block the reengagement of the heads. Notably, the inhibition of the ATPase activity doesn’t differentiate between no ATP binding and inhibition of ATP hydrolysis.

### CIP3 induces precocious separation of sister chromatids in cells

**Figure 4:**
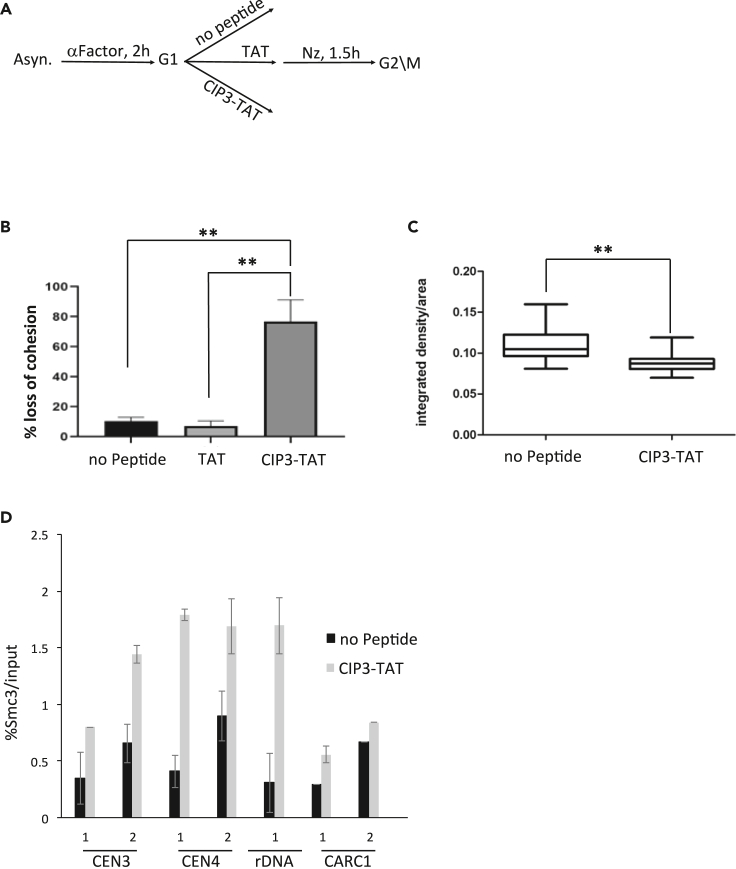
CIP3 inhibits sister chromatid cohesion and chromosome condensation (A) Flowchart outlining the sister chromatid cohesion assay. (B) yME-961 strain cells were grown and treated with peptides, as shown in A. Premature sister chromatid separation was determined by counting the number of cells showing two GFP dots. At least 300 cells were counted in 3 independent experiments. ∗∗p < 0.001. (C) Cells were grown to mid-log phase. The culture was divided into two flasks, one being treated with CIP3-TAT. Condensation was determined in cells at the G2/M phase by 2-photon microscopy. ∗∗p < 0.001. (D) yKS-008 strain (Smc3-V5) cells were grown and treated with CIP3-TAT peptide, as shown in A. Cells were processed for ChIP with antibodies against V5.

The formation of Smc3/CIP3 complexes supports our preliminary conclusion that CIP3 inhibits cohesin activity in cells. Aiming to improve the stability of the peptide in cells, we switched to using an *in vitro* synthesized peptide. This approach has been successfully used to inhibit protein-protein interactions in both yeast and mammalian cells.^26^^,^^38^^,^^39^^,^^40^^,^^41^ Previous reports showed that peptide delivery into cells increased by fusing them into a 11-amino acid peptide originating from the HIV TAT protein.^42^^,^^43^^,^^44^ We validated peptide entry into yeast by incubating a 26-amino acid control peptide (ContP), which was fused to the TAT, and a fluorescein isothiocyanate (FITC) fluorophore. Thereafter, 10 mM of the ContP was added to yIO-001 strain cells grown to mid-log phase at 30°C. The cells were incubated with the peptide for 1 h and analyzed by epifluorescence microscopy. No autofluorescence was found in the control cells that were not exposed to the peptide, which was in contrast to the emission from cells incubated with the ContP peptide (Figure S5. Delivering peptides into yeast cells, related to Figure 4).

The potential of CIP3-TAT to inhibit cohesion activity was explored by analyzing sister chromatid cohesion in cells arrested at the G2/M. Inserted in the LYS4 locus were LacO arrays, which bind LacI-GFP. In unperturbed cells arrested at the G2/M phase of the cell cycle, cohesin tethers the sister chromatids visualized under the microscope as a single GFP dot. When cohesin is inactivated, the sister chromatids fall apart and are seen as two distinctive GFP dots. yME-961 strain cells were grown to mid-log phase and arrested at the G1 phase of the cell cycle. Cells were divided into three samples, of which two were supplemented with TAT peptide alone or CIP3-TAT for 1 h. Then, the cells were released into the cell cycle until their rearrest at the G2/M phase. Cells were processed for cohesion assay (Figure 4A). No cell cycle delays were found between the samples (Figure S6, Flow cytometry of cells arrested at different cell cycle stages, related to Figure 4). Untreated cells and TAT-treated cells revealed normal sister chromatid cohesion levels. However, approximately 80% of the CIP3-TAT treated cells revealed precocious separation of the sister chromatids (Figure 4B).

Chromosome condensation depends on cohesin. However, this cohesin function can be separated from sister chromatid cohesion activity.^45^^,^^46^ We tested this possibility by analyzing chromatin in G2/M cells via two-photon microscopy.^47^^,^^48^ Cells were grown and treated as before. The condensation level in TAT-treated cells was indistinguishable from wild-type cells. However, in CIP3-TAT-treated cells, chromatin was decondensed in comparison with the untreated cells (Figure 4C). Altogether, the results indicate that CIP3-TAT penetrates live cells and inhibits cohesin activity.

### Smc1/Smc3 head engagement affects cohesin chromosomal residency

To test the effect of CIP3 on the association of cohesin with chromatin, we performed a chromatin immunoprecipitation (ChIP) assay. yKS-008 strain (Smc3-V5) cells were grown to mid-log phase and arrested in the G1 phase of the cell cycle. Cells were untreated or supplemented with CIP3-TAT and then released into the cell cycle until they were rearrested at the G2/M phase. Cells were processed for ChIP with antibodies against V5 (Figure 4D). Cohesin residency was explored on loci on which cohesin enrichment: the centromeres of chromosome 3 and chromosome 4, the rDNA locus on chromosome 12 and the cohesin-associated region C1 (CARC1) on chromosome 3. Interestingly, cohesin residency increased in cells treated with CIP3-TAT in all tested regions. This result implies that head domain engagement is not essential for cohesin loading or chromosomal binding. However, it stabilizes non-cohesive chromosome-bound cohesin. Our results that cohesin residency on chromatin increased is in agreement with previous studies that showed that inhibition of the ATP hydrolysis by mutations or using ATP analogs. Thus, we conclude that CIP3 inhibits ATP hydrolysis rather than blocks ATP binding and cohesin loading.

### Mitotic delay in CIP3-treated human cells

**Figure 5:**
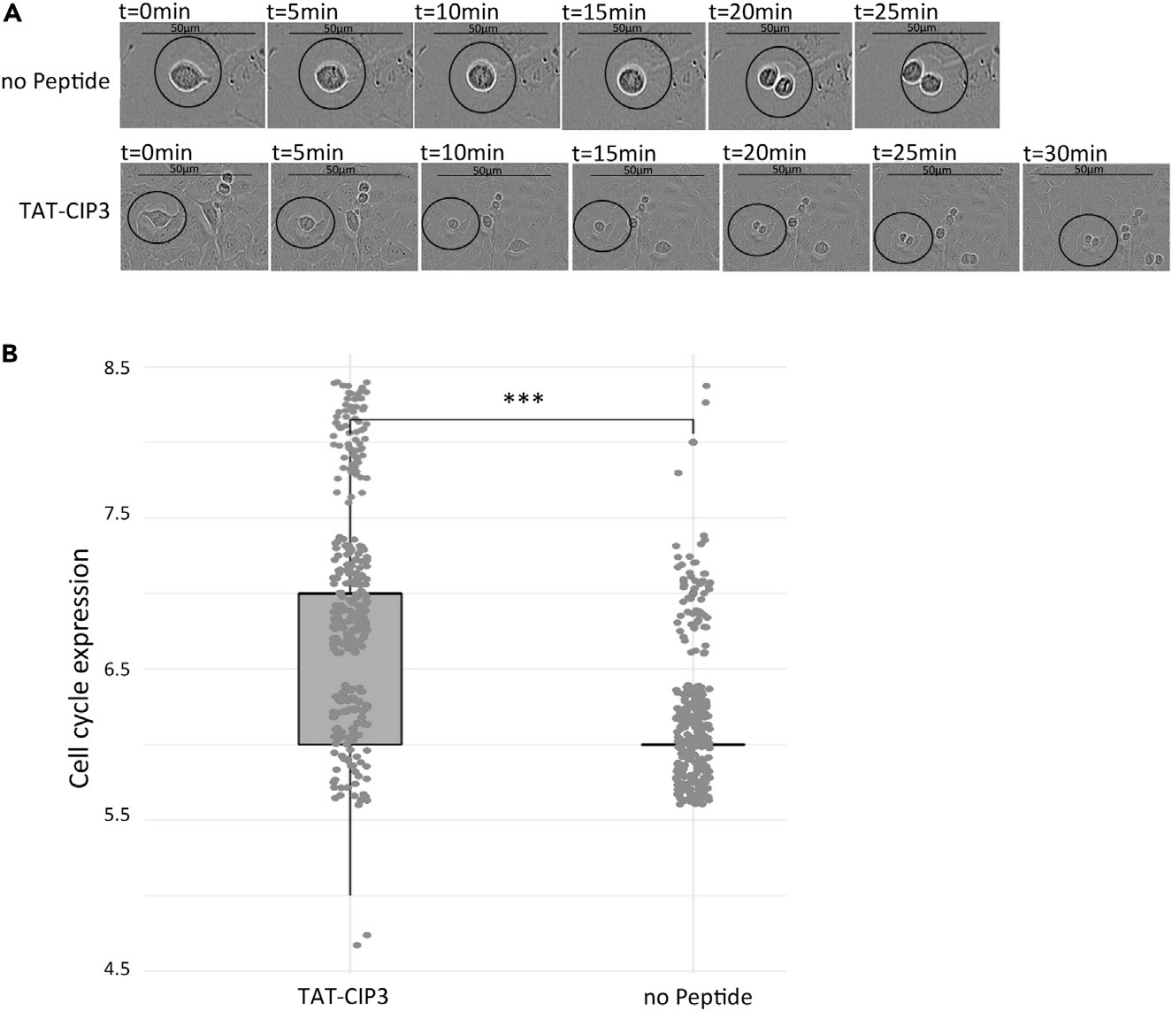
CIP3 causes a mitotic delay in human cells U2OS cells were plated in a 96-well plate and grown for 20 h to allow adherence. CIP3 peptide was added to the growth medium, and cells were grown for additional 20 h. Images were taken every 5 min. Mitosis length was determined by counting the number of images in which mitotic chromosomes were visualized. (A) Representative image. (B) Quantitation of mitosis length in untreated and CIP3-TAT treated cells. At least 300 cells were counted for each condition. ∗∗∗p < 0.0001.

CIP3 was derived from a highly conserved region of Smc1 (Figure 1D). Therefore, we sought to test its ability to affect cohesin activity in human cells. U2OS cells were diluted into a 96-well plate and grown for about 20 h. Added to this was 10 nM peptide and the cells were grown for another 20 h. Images of the cells were taken every 5 min (Figure 5A). We expected cohesion loss to result in a mitotic delay. We calculated mitosis time starting from the first image in which condensed mitotic chromosomes were visualized until their disappearance at mitosis exit. Strikingly, the average mitosis time in CIP3-TAT treated cells increased by about 10% (from 6 to 6.5 min), indicating a mitotic delay (Figure 5B). This result suggests that CIP3-TAT inhibits cohesin in both yeast and mammalian cells.

## Discussion

Cohesin is a sophisticated molecular machine essential for chromosome segregation in dividing cells. The different subunits orchestrate the organization of chromatin fibers into higher-order structures that are essential for maintaining genome stability and function.^2^^,^^3^^,^^4^ Cohesin activity depends on its ability to adopt different conformations and shifts between them in response to molecular signals.^11^^,^^16^^,^^17^^,^^18^^,^^19^ The energy for this movement comes from ATP hydrolysis in the two ABC-type ATPase domains in the SMC heads. Smc1 and Smc3 head engagement is the immediate response to ATP binding to the SMCs,^10^^,^^11^^,^^12^^,^^13^^,^^14^^,^^15^^,^^16^ yet the role of the head interaction remained elusive.

Based on rational design, we developed a peptide, CIP3, derived from the Smc1 interaction surface that interacts with the Smc3 head domain. To our best knowledge, this is the first development of a specific cohesin inhibitor. We showed that the peptide CIP3-TAT binds Smc3 (K_d_ 56 nM). Remarkably, the binding affinity of the peptide to Smc3 is reduced 10-fold in the presence of ATP (K_d_ 544 nM). We note that binding kinetics was measured with purified protein of Smc3 head domain. These affinities might be modified if the holocomplex was used. However, this is a technically challenging experiment that is beyond the scope of this work. Nevertheless, the results indicate that the unbound conformation of Smc3 is favored for binding of the CIP, while ATP binding reduces binding affinity to the peptide, most likely due to the conformational change of the protein in the ATP-bound state.^19^ Biochemical measurements of the ATPase rate of the cohesin holocomplex revealed inhibition of the activity by the peptide. This result can explain in two ways. The binding of ATP to Smc3 is blocked, or ATP hydrolysis is inhibited as the Smc1 hydrolysis site is mislocalized and thus fails to form a fully active site. As previously demonstrated, ATP binding is essential for cohesin loading onto the chromatin.^10^ The peptide does not compromise ATP binding, as the ChIP analysis revealed that cohesin is bound to chromatin. However, other studies showed that ATP hydrolysis is also important to cohesin release from the chromatin.^11^^,^^21^^,^^49^ These opposite functions were explained by asymmetric ATPase by the two sites harbored in the head domain. The results suggest that the Smc1 Walker B domain is not essential for DNA entrapment but involved in relaying the hydrolysis signal and transforming cohesin to its cohesive state.^21^ The model we described in Figure 2A suggests that the peptide blocks the Walker A. We suggest that the peptide competes with Smc1 and partially or fully inhibits head engagement. This blocking prevents cohesin tethering activity, as shown previously.^11^^,^^13^^,^^21^^,^^49^ Therefore, proper head alignment is essential to ATP hydrolysis rather than ATP binding. Thus, our work provides a molecular explanation to the previous studies suggesting that the Smc1 Walker B mutations compromise head engagement. Full dynamics models of the CIP3 interaction with Smc3 will give further insights into the inhibition mechanism.

The structural basis of cohesin activity involves a shifting conformation that defines SMC (S) and kleisin (K) compartments located in the SMC coiled-coil lumen and between the heads and the kleisin, respectively.^18^ Initially, the DNA enters the S compartment while head engagement following ATP binding entraps the DNA in the K compartment. Upon ATP hydrolysis, a conformational change induces a juxtaposition of the heads. Our results imply that the peptide does not compromise the formation of the K compartment as cohesin binds to the chromosome. However, the peptide inhibits ATP hydrolysis-dependent head disengagement, inhibiting Smc3 conformational change and cohesion-tethering activity. Cohesin binding to chromatin is likely mediated by a non-topological mechanism.^15^^,^^50^^,^^51^

In addition to cohesin mitotic functions, the complex is important for chromatin organization during interphase.^52^^,^^53^^,^^54^ Loop extrusion is the process by which chromatin threads through cohesin to form loops and other higher-order structures such as topologically associated domains (TADs).^55^^,^^56^^,^^57^ These cohesin-mediated organizations play roles in the regulation of replication, transcription, and DNA repair. Here, we examined the effect of CIP3 on cohesin mitotic functions. However, loop extrusion relay on continuous ATPase activity cycles that drive the threading.^56^ Therefore, we expect that CIP3 will have a significant effect on chromatin organization interphase and the associated genome process. A future goal of our work is to determine this effect in yeast and human cells.

We spotlighted the head domain because interactions therein are dynamic. As a result, the interaction surface in Smc3 is occasionally exposed, allowing the peptide to compete with the native protein. Other cohesin interactions that may fulfill this requirement include the hinge-coiled coil and Smc3-Eco1.^17^^,^^23^^,^^58^^,^^59^ These regions are future targets for the development of PPI-inhibiting peptides. Similarly, the same regions in the related SMC complexes, condensin, and SMC5/6 can be used as targets and may evolve as essential tools for the dissection of the molecular mechanism of SMC complexes.

Peptides are gaining increasing attention as therapeutics. Currently, approximately 100 peptide drug products are marketed in the United States, Europe, and Japan, treating a wide range of indications. Furthermore, peptides are ideal candidates for the inhibition of PPIs because they can mimic a protein surface to effectively compete for binding.^28^^,^^60^^,^^61^ We have reported on the first development of a peptide that inhibits cohesin. Initially, we tried to screen for the inhibiting activity by expressing the peptide in yeast cells from a plasmid. This approach has the potential to enhance the development of PPI-inhibiting peptides. However, we detected a weak phenotype that was not improved by extending mRNA length via a T2A vector. Other genetic and molecular modifications may be needed to enhance phenotypic outcomes before this approach can be widely adopted for screening peptide activity.

CIP3 was designed based on the sequence of *S. cerevisiae* proteins. This region in the protein is conserved between yeast and human proteins (Figure 1E). Significantly, we showed that treating human cells with the peptide leads to mitotic delay. This suggests that the peptide inhibits cohesin in these cells, as well. Showing a cohesion effect is a technical challenge, given the high concentration of peptides needed to grow enough cells for chromosome analysis. Our findings show that this approach is useful for inhibiting cohesin in both yeast and human cells. This direction may have therapeutic potential in the treatment of cancer as cohesin inhibition in cancer cells could arise as a new strategy to inhibit cell proliferation. A remaining challenge will be targeting the peptides to the tumor. Several studies in this direction identified leader sequences that can provide this specificity.^62^ An additional future objective is peptidomics, which aims to improve the affinity and cellular stability of the synthetic peptide in the cell. All of these are future goals for our labs.

### Limitations of study

Peptides are often unstable in cells. We assume that CIP3 instability is associated with the relatively weak phenotypes observed in cellular experiments, which differ from the strong phenotypes observed in the biochemical and molecular assays. Peptidiomimic is expected to improve the cellular stability of the peptide. We present a model of the Smc3-CIP3 complex. In order to achieve a comprehensive understanding of the Smc3-CIP3 interaction, experimental validation of the model and kinetics studies are required. We have demonstrated that CIP3 inhibits cohesin’s ATPase activity and induces premature separation of the sister chromatid in the G2 phase of the cell cycle. It would be interesting to explore the effect of CIP3 on loop extrusion, as it affects interphase chromatin organization and may result in transcription misregulation and misfiring of replication origins.

## STAR★Methods

### Key resources table

**Table:** 

REAGENT or RESOURCE	SOURCE	IDENTIFIER
**Antibodies**		

Mouse anti-HA (12CA5)	Roche	CAT# 11666606001; RRID:AB_514506
Mouse anti-V5	Invitrogen	CAT# R960-25; RRID:AB_2556564
Rabbit anti-V5	Millipore	CAT# AB3792; RRID:AB_91591
Rabbit anti-mCherry	Abcam	CAT# ab183628; RRID:AB_2650480
Rabbit anti-GFP	Abcam	CAT# ab290; RRID:AB_303395
Rat anti-tubulin	Abcam	CAT# ab6160; RRID:AB_305328

**Bacterial and virus strains**		

*E. coli* BL21 (DE3)	Bio-Lab	959758026610

**Chemicals, peptides, and recombinant proteins**		

MKQNEQLHVEQLSGGQKTV	This paper	CIP1
MIIGSNGSGKSN	This paper	CIP2
MKRFKDMEYLSGGEKT	This paper	CIP3
KRFKDMEYLSGGEKT-GGYGRKKRRQRRR	This paper	TAT-CIP3
FITC-SGYGRKKRRQRRR-GGGLNPYWMETFT	This paper	ContP
Smc3 head domain	This paper	Smc3hd
Cohesin (Smc1/Smc3/Mcd1/Scc3)	Frank Uhlmann^49^	Cohesin
Loader (Scc2/Scc4)	Frank Uhlmann^49^	Loader

**Critical commercial assays**		

Phosphate Assay Kit - PiColorLockTM 600	Abcam	ab270004
Western Antares	Cyanagen	XLS142,0250
Maxtract high-density columns	Quiagen	129056

**Experimental models: Cell lines**		

Human U2OS cells	ATCC	HTB-96; RRID:CVCL_0042

**Experimental models: Organisms/strains**		

*Saccharomyces cerevisiae* strain A364A	ATCC	208526

**Oligonucleotides**		

Please see SI Table S3	Sigma-Aldrich	

**Recombinant DNA**		

pGAL-CIP2	This paper	pME2
pGAL-CIP3	This paper	pME3
pGAL-CIP1	This paper	pME4
pGAL-SCC4	The Onn Lab^63^	pIO-014
pGAL-GFP-TAT-mCherry	This paper	pME-012
pGAL-GFP-TAT-CIP3	This paper	pAM-84

**Software and algorithms**		

HPEPDOC 2.0 server	Huang Laboratory^32^^,^^33^^,^^34^^,^^35^	http://huanglab.phys.hust.edu.cn/hpepdock/
ImageJ	NIH^64^	https://imagej.nih.gov/ij/

**Other**		

Inverted confocal microscope LSM780	Zeiss	N/A
Incocyte SX5	Sartorius	N/A
automated peptide synthesizer	Syro I, Biotage	N/A
HPLC 1260 Infinity II LC Systems	Agilent	N/A
Luna C18(2)	Phenomenex	112926-00-8
Matrix-assisted laser desorption/ionization mass spectrometry (MALDI-MS)	Bruker	N/A
Field-Effect Biosensing (FEB) Agile R100	Nanomedical Diagnostics	N/A
EmulsiFlex®-C3, a high-pressure homogenizer	Avestin	N/A
Monolith NT.115	NanoTemper	N/A
AKTA Avant 25	Bio-Rad	N/A
Superdex 200 Increase 10/300 GL	Cytivia	28-9909-44
HiTrap Heparin HP	Cytivia	17-0406-01
Superose 6 Increase 10/300 GL	Cytivia	29-0915-96
Monolith NT.115	NanoTemper	N/A
Monolith NT.115 standard Treated capillaries	NanoTemper	K002
Bullet blender	Next Advance	N/A
PVDF membrane	Millipore	A4612
LAS4000	GE	N/A

### Resource availability

#### Lead contact

Information and requests for resources should be directed to and be fulfilled by the Lead Contact, Itay Onn (Itay.Onn@biu.ac.il).

#### Materials availability

All materials generated in this study are available on request to the lead contact.

### Experimental model and subject details

#### Plasmids, primers, yeast strains, cell growth, and synchronization

The yeast strains, plasmids, and primers used in this study are listed in Table S1. Yeast strains, S2. Plasmid list, and S3. Primer list, respectively. Yeast was grown in YPD or SD-URA media. Cells were arrested in the G1 phase by adding 1.5 x 10^-8^ M alpha-factor (Zymo Research) and released by washes with 0.1 mg/ml pronase E (Sigma). G2/M arrest was achieved by supplementing the growth medium with 15 μg/ml nocodazole (Sigma).

#### Human cell line

U2OS cells were maintained in Dulbecco's Modified Eagle Medium (DMEM) (Gibco) with 10% fetal bovine serum (FBS) (Corning), 2 mM l-glutamine (Gibco), 1% penicillin-streptomycin (Gibco), and 1 mM sodium pyruvate (Gibco), at 37°C, 5% CO_2_. In a 96-well plate, 9x10^4^ cells were plated and grown for 20 hours to allow adherence. The peptide was added to the growth medium, and cells were grown for additional 20 hours in an Incucyte® SX5 Live-Cell Analysis Instrument (Sartorius). Images were taken every 5 minutes. The significance between conditions was determined by using Student's t-test.

### Method details

#### Smc3 head domain purification

The Smc3 head domain was cloned into pET28b as described in^36^ to create pCT18. In brief, the sequence encoding Smc3 N′ terminal (amino acids 2-204) and C′ terminal (amino acids 1001-1230) separated by 13 amino acids linker ESSKHPTSLVPRG were cloned into Eco1-XhoI sites. A his-tag was added to the N-terminus of the protein. pCT18 was expressed in 2 L of E. coli BL21 by adding 1 mM isopropyl -D-1-thiogalactopyranoside (IPTG) for 16 hours at 16°C. Cells were harvested by centrifugation, frozen in liquid N_2,_ and kept at -80°C. The cell pellet was thawed on ice and resuspended in lysis buffer (44.8 mM Na_2_HPO_4_, 5.2 mM NaH_2_PO_4_, 150 mM NaCl, 5 mM β-mercaptoethanol, and protease inhibitor cocktail (Sigma)). Cells were lyzed in an EmulsiFlex®-C3, high-pressure homogenizer (AVESTIN). The lysate was clarified in an Avanti high-speed centrifuge (Beckman Coulter) fitted with a JA-25.50 rotor at 10,000 rpm for 1 hour at 4°C. The lysate was added to 1 ml Ni-NTA agarose beads and incubated at 4°C for 2 hours. The beads were packed into a column and washed with 10 ml of 44.8 mM Na_2_HPO_4_, 5.2 mM NaH_2_PO_4_, 150 mM NaCl, 5 mM β-mercaptoethanol, and 20 mM imidazole. Elution from the column was performed by washing it with 4 ml of 44.8 mM Na_2_HPO_4_, 5.2 mM NaH_2_PO_4_, 150 mM NaCl, 5 mM β-mercaptoethanol, and 500 mM imidazole. The imidazole in the eluate was removed by overnight dialysis at 4°C against a lysis buffer, and the protein was repurified on Ni-NTA, as above. Protein aliquots were frozen in liquid N_2_ and kept until use at -80°C.

#### Field-effect biosensing

The binding kinetics of cohesin-inhibiting peptide 3 (CIP3) to Smc3 were measured by field-effect biosensing (FEB) Agile R100 label-free binding assay (Cardea), following their standard protocol, and as we have done previously,.^65^^,^^66^^,^^67^ Briefly, 500 nM of CIP3 was immobilized on a graphene sensor chip by functionalizing the amine groups on the sensor surface. To establish the baseline of the current for the experiment, we used phosphate-buffered saline (PBS). Next, PBS was aspirated, and the changes in the baseline current were monitored in real-time, using 20 μM, 50 μM, 100 μM, 200 μM, 500 μΜ, 1000 μM, 1500 μM, 2000 μM, and 4000 μM of purified Smc3 head domain. K_d_ values were calculated using DataLINE 2.0 software by applying either a Hill equation fit or using k_on_ and k_off_ values at a single concentration. The K_d_ values obtained by these two methods were almost identical.

#### Co-immunoprecipitation and Western blot are described in.

Co-immunoprecipitation and Western blot are described in.^46^^,^^63^^,^^68^^,^^69^ Cells were grown to mid-log phase, pelleted and washed with dH_2_O, and frozen in liquid nitrogen. Pellets were resuspended in 350 μl IP50/150 buffer (50 mM Tris, pH 8.0, 50/150 mM NaCl,1 mM EDTA, 5 mM MgCl2, 10% glycerol, 0.4% NP-40, protease inhibitor cocktail (Sigma)). For Smc3 acetylation experiments, IP50 was supplemented with 10 mM sodium butyrate (Sigma). Cells were lysed by adding glass beads (Sigma) to the resuspended pellets, followed by 4 working cycles of 1 minute in a bullet blender (Next Advance). The lysates were cleared by two centrifugations of 5 and 15 min at 15,000 ×g at 4°C. Immunoprecipitations were performed at 4°C, and the appropriate antibodies were added for 1 h. The antibodies were collected on protein A magnetic beads (Bio-Rad) 1 h later and washed 3 times with IPH50/IPH150 and resuspended in 32 μl Laemmli buffer. Standard procedures for sodium dodecyl sulfate-polyacrylamide gel electrophoresis and Western blotting were followed to transfer proteins from gels to a polyscreen PVDF membrane (Millipore). Membranes were blotted with the primary antibodies. Antibodies were detected using Western Antares (Cyanagen) and LAS 4000 (GE). Antibodies used in this study were: mouse anti-HA (Roche), mouse anti-V5 (Invitrogen/Millipore), rabbit anti-mCherry (Abcam), rabbit anti-GFP (Abcam), and rat anti-tubulin (Abcam).

#### Peptide design, synthesis, and delivery to yeast

CIP3 was designed based on rational design as previously described.^70^^,^^71^^,^^72^^,^^73^ Peptides were chemically synthesized using a fully automated peptide synthesizer (Syro I, Biotage) on solid support by following the solid-phase peptide synthesis (SPPS) methodology^74^ using the fluorenyl-methoxycarbonyl (Fmoc)/tert-butyl (tBu) protocol. Final cleavage and side-chain deprotection were done manually. The peptides were analyzed using analytical reverse-phase high-pressure liquid chromatography (RP-HPLC) (1260 Infinity II LC System, Agilent, CA, USA) and matrix-assisted laser desorption/ionization mass spectrometry (MALDI-MS) (autoflex® maX, Bruker, Billerica, MA, USA), and purified by preparative RP-HPLC (1260 Infinity II LC System, Agilent, CA, USA). The full description of peptide synthesis is provided in the supplementary information. In yeast experiments, the peptide was added to G1- arrested cells for 1 hour before they were released into the cell cycle.^75^^,^^76^ A detailed protocol for peptide synthesis and purification will be sent upon request.

#### Structural modeling

The coordinates of the Smc3 structure (PDB 4ux3) and the CIP3 sequence (MKRFKDMEYLSGGEKT) were uploaded to the HPEPDOC 2.0 server,^32^^,^^33^^,^^34^^,^^35^ for flexible peptide-protein docking receptor and peptide inputs, respectively. No binding sites were specified in Smc3 or CIP3 and the docking parameters were set to default.

#### Cohesin purification and ATPase assay

Cohesin and the Scc2-Scc4 loader were expressed in S. cerevisiae as described in^37^ with the following modifications: Cells were resuspended in buffer A (50 mM Hepes-NaOH pH 7.5, 300 mM NaCl, 2 mM MgCl_2_, 20% (vol/vol) glycerol, 0.5 mM Tris(2-carboxyethyl)phosphine hydrochloride (TCEP), 0.5 mM Pefabloc (Sigma-Aldrich), and a protease inhibitor cocktail (Sigma-Aldrich) and lysed by seven continuous passes in EmulsiFlexby-C3 (Avastin) at 4°C. The lysate was clarified, and purifications were continuous, as described in.^37^

ATPase assay has been done with the piColorLock kit (Expedeon) according to the manufacturer manual. 10nM cohesin was mixed with a 20 nM loader and 3.3 mM dsDNA with or without 20 mM TAT-CIP3. The reaction mixture was pre-incubated on ice for 5 minutes. 0.25 mM ATP was added to start the enzymatic reaction. The reaction was incubated at 30°C for 20 minutes and the amount of free was measured for 30 min minutes by absorbance in 650 nm every 5 min for 50 minutes.

#### Microscale thermophoresis (MST)

Yeast strain yAM-945 cells containing Smc3-GFP were grown in SD-URA galactose to mid-log phase. Cells were collected by centrifugation and washed with water. The cell pellet was resuspended in 350 μl of MST Buffer (NanoTemper) supplemented with 0.05% Tween-20 (Sigma). Glass beads were added, and cells were lyzed by 4 cycles of 1 minute in a bullet blender (next advance). The protein extract was clarified by centrifugation at 1000 g for 5 minutes. 20 nM total cellular protein concentration was mixed with 5 μM of CIP3-TAT and loaded into Monolith NT.115 standard Treated capillaries (K002) (NanoTemper). The formation of the CIP3-TAT and cohesin complex was analyzed by Monolith NT.115 (NanoTemper) in the binding mode that detects the formation of complexes without calculating the binding constants.

#### Cohesion dot assay, chromatin immunoprecipitation (ChIP)

The cohesion GFP dot assay and ChIP are described in.^46^^,^^63^^,^^68^ In brief, cohesion was studied by visualizing LacO arrays inserted at the LYS locus in a strain expressing LacI-GFP. Cell cultures were grown in a YEPD to OD600=0.5 and synchronized by nocodazole. Cells were fixed by incubating with 100 μl of 4% paraformaldehyde, 15min at room temperature and washed once (5,000rpm, 30sec, room temperature) with 1ml KOP_4_/sorbitol solution (0.1 M KOP_4_ pH=7.5 with 1.2 M sorbitol), resuspended in 100 μl of KOP_4_/sorbitol and stored at 4°C up to one month. Cells were viewed by using the Zeiss inverted Cell Observer microscope.

Crosslinking for ChIP was done by adding 1% formaldehyde to nocodazole arrested cell culture. The DNA was sheared by sonication Bioruptor® Plus. Immunoprecipitation was performed using the V5 antibody followed by AG agarose beads. DNA was purified with phenol: chloroform: isoamyl alcohol (25:24:1) method using the Maxtract high-density columns (Quiagen). Precipitated DNA was analyzed by qPCR with the primers listed in Table S3.

#### Condensation assay by two-photon microscopy

The method is described in.^47^^,^^48^ Briefly, cells were grown to mid-log phase. Two-photon microscopy of live cells was performed with the LSM780 (Zeiss) inverted confocal microscope, using the Chameleon Vision II (Coherent) multiphoton laser (3 W, pulse width 140 fs at peak, repetition rate 80 MHz, tuning range 680-1080 nm, excitation 768 nm/image collection 512 nm). Slides were visualized with X63 N/A 1.4 objective lenses. Cells in G2/M were selected based on their morphology. Images were analyzed using Image J processing software. The condensation level is represented by the division of the nuclear-integrated density by its circumference. The significance between conditions was determined by using Student's t-test.

### Quantification and statistical analysis

All Statistical analyses were performed in GraphPad Prism 8 or Excel. Data are presented as means ± SEMs. Comparisons of the two groups were analyzed by Student’s t-test. All experiments reported in the paper were repeated three times. At least 300 cells were counted in each cohesion assay. For the two-photon microscopy condensation, at least 40 nuclei were analyzed.

## Data Availability

• All data has been included in main figures or supplemental information. All data reported in this paper will be shared by the lead contact upon request. • This paper does not report original code. • Any additional information required to reanalyze the data reported in this paper is available from the lead contact upon reasonable request.
