# Supplementary material for: Developing a peptide to disrupt cohesin head domain interactions
Source: iScience. 2023 Jul 28;26(9):107498. doi: 10.1016/j.isci.2023.107498 (PMC10470313; doi:10.1016/j.isci.2023.107498)
Supplement: Document S1. Figures S1–S6 and Tables S1–S5 [file mmc1.pdf]

iScience, Volume *26*

Supplemental information

Developing a peptide to disrupt cohesin head domain interactions

Maria Elias, Samar Gani, Yana Lerner, Katreen Yamin, Chen Tor, Adarsh Patel, Avi Matityahu, Moshe Dessau, Nir Qvit, and Itay Onn

Supplemental Information

Supplementary Figure Legend

Supplementary Figure S1. Peptide expression from a plasmid containing T2A ribosome-skipping sequence, Related to Figure 2. A. Schematic of protein expression from mRNA containing two open reading frames separated by T2A sequence. **B**. Cells were transformed with pME12 (pGAL-GFP-T2A-mCherry). Cells were grown in SC-URA/raffinose to mid-log phase. The culture was divided in two, with one of the flasks being supplemented with galactose. Cells were grown for an additional 2 hours, fixed, and analyzed by epifluorescence microscopy. **C**. The cells described in B were lyzed. The cell extracts were analyzed by Western blot analysis with anti-GFP and anti-mCherry antibodies. **D.** Schematic of CIP3 cloned into pGAL in tandem with GFP and separated by T2A sequence. The arrows indicate the location of the primers used in E. **E.** Fold-change of mRNA of CIP3-T2A-GFP mRNA in cells expression was induced with galactose in comparison to uninduced cells grown in raffinose.

Supplementary Figure S2. Purification of CIP3-TAT and Smc3 head domain, Related to Figure 3. A. CIP3 was synthesized as described in Materials and Methods and Supplementary Information. HPLC analysis of the peptide after purification is shown. **B.** Mass spectrometry analysis of the purified CIP3-TAT. The expected CIP3-TAT mass of 3447 Da was detected. **C.** Smc3 head was purified as described in Materials and Methods. FT=flowthrough, W1 and W2=washes, E=elution. **D.** The elution fraction of the Smc3 head after the second purification cycle on Ni-NTA agarose column. The eluted proteins were analyzed by SDS-gel electrophoresis stained with Coomassie. The purified Smc3 head domain is indicated by the arrow.

Supplementary Figure S3. Binding of CIP3-TAT to cohesin by microscale thermophoresis, Related to Figure 3. 10 μM CIP3-TAT was added to protein extracts from strain yAM-945 (Smc3-GFP). The formation of the CIP3-TAT and cohesin complex was analyzed by MST in binding mode. **A.** MST trace. **B.** Binding analysis.

Supplementary Figure S4. Purification of cohesin holocomplex and loader, Related to Figure 3. Cohesin holocomplex (Smc1-Smc3-Scc1-Scc3) and the loader (Scc2-Scc4) were expressed and purified as described in Material and Methods. The purified proteins were analyzed on gel, followed by coomassie staining. **A.** The purification of cohesin holocomplex on IgG (lane 1-lysate, 2-flowthrough, 3-wash, 4-elution/heparin input) followed by heparin column (lanes 5-7 are three constitutive elution fractions containing cohesin). **B.** The purification of the loader on IgG (lane 1-lysate, 2-flowthrough, 3-wash, 4-elution/heparin input) followed by heparin column (lanes 5-7 are three constitutive elution fractions containing cohesin)

Supplementary Figure S5. Delivering peptides into yeast cells, Related to Figure 4. Strain yIO-001 cells at mid-log phase were treated with 10 mM peptide ContP labeled with FITC for 1 hour at 30 °C. The entry of the peptide into the cells was analyzed by epifluorescence microscopy.

Supplementary Figure S6. Flow cytometry of cells arrested at different cell cycle stages , Related to Figure 4. The cell cycle stage of the cells, described in Fig. 4B, was determined by flow cytometry. Asyn.=Asynchronous cells.

Figure S1

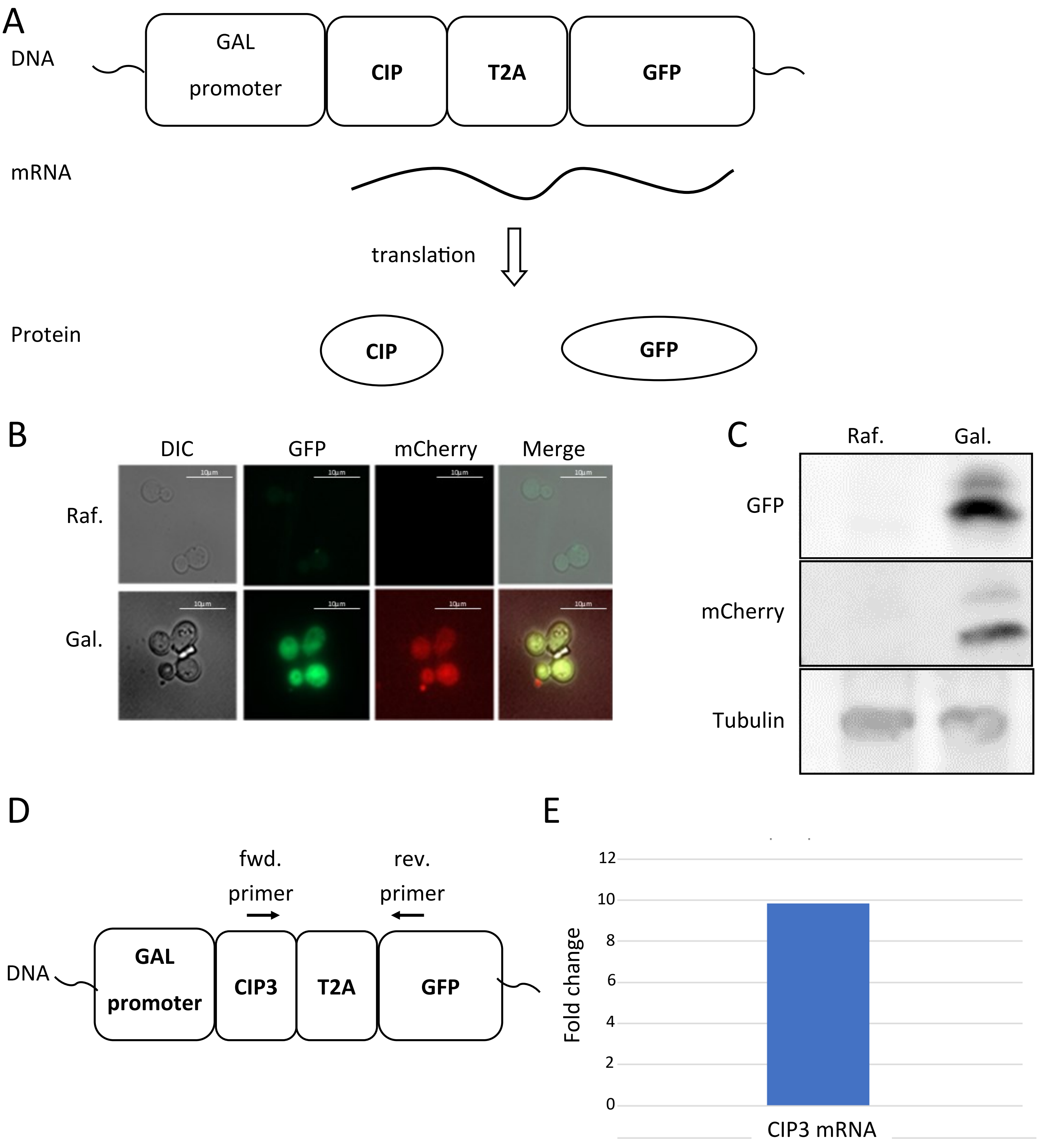
A
DNA
GAL
promoter
CIP
T2A
GFP
mRNA
translation
Protein
CIP
GFP
B
DIC
GFP
mCherry
Merge
Raf.
Gal.
C
Raf.
Gal.
GFP
mCherry
Tubulin
D
fwd.
primer
rev.
primer
DNA
GAL
promoter
CIP3
T2A
GFP
E
12
10
8
6
4
2
0
Fold change
CIP3 mRNA

Figure S2

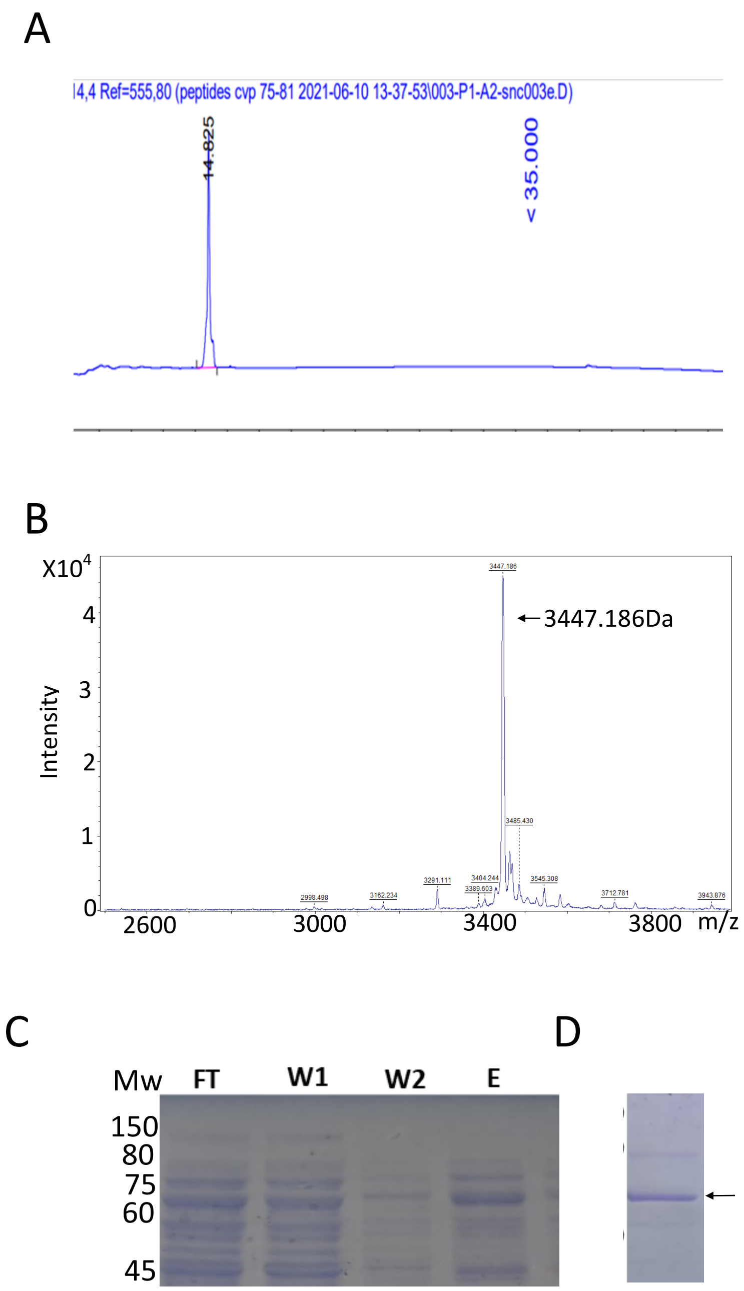
A
l4,4 Ref=555,80 (peptides cvp 75-81 2021-06-10 13-37-53\003-P1-A2-snc003e.D)
14.825
< 35.000
B
X10^4
Intensity
4
3
2
1
0
3447.186
3447.186Da
3485.430
3291.111
3404.244
3389.603
3545.308
3712.781
3943.876
2998.498
3162.234
2600
3000
3400
3800
m/z
C
Mw
FT
W1
W2
E
150
80
75
60
45
D

Figure S3

A

B

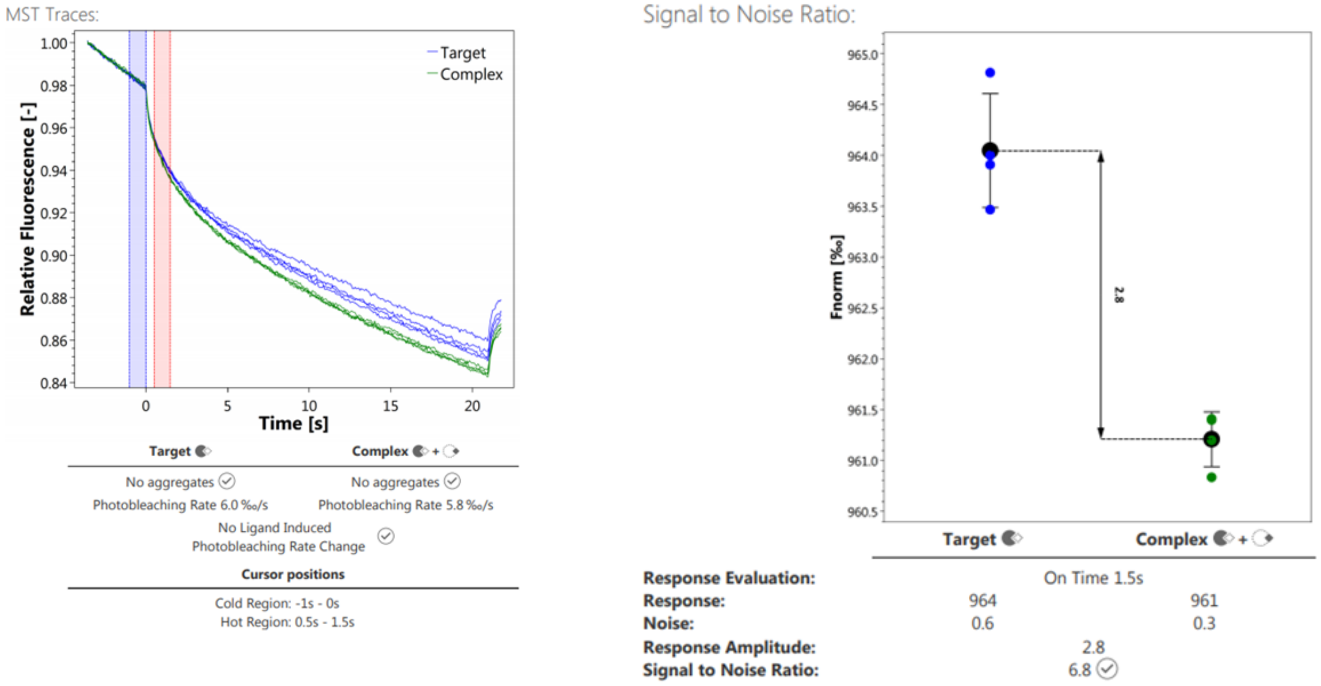
MST Traces:
Relative Fluorescence [-]
Time [s]
Target
Complex
Target
Complex
No aggregates
Photobleaching Rate 6.0 ‰/s
No aggregates
Photobleaching Rate 5.8 ‰/s
No Ligand Induced Photobleaching Rate Change
Cursor positions
Cold Region: -1s - 0s
Hot Region: 0.5s - 1.5s
Signal to Noise Ratio:
Fnorm [‰]
2.8
Target
Complex
Response Evaluation: On Time 1.5s
Response: 964 961
Noise: 0.6 0.3
Response Amplitude: 2.8
Signal to Noise Ratio: 6.8

Figure S4

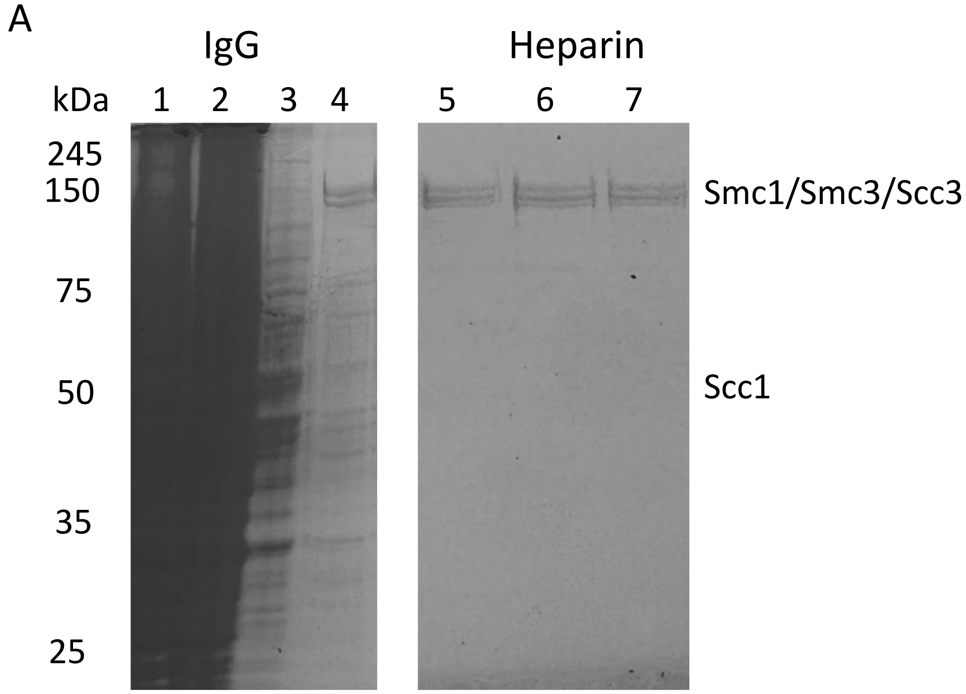
A
IgG
Heparin
kDa
1
2
3
4
5
6
7
245
150
75
50
35
25
Smc1/Smc3/Scc3
Scc1

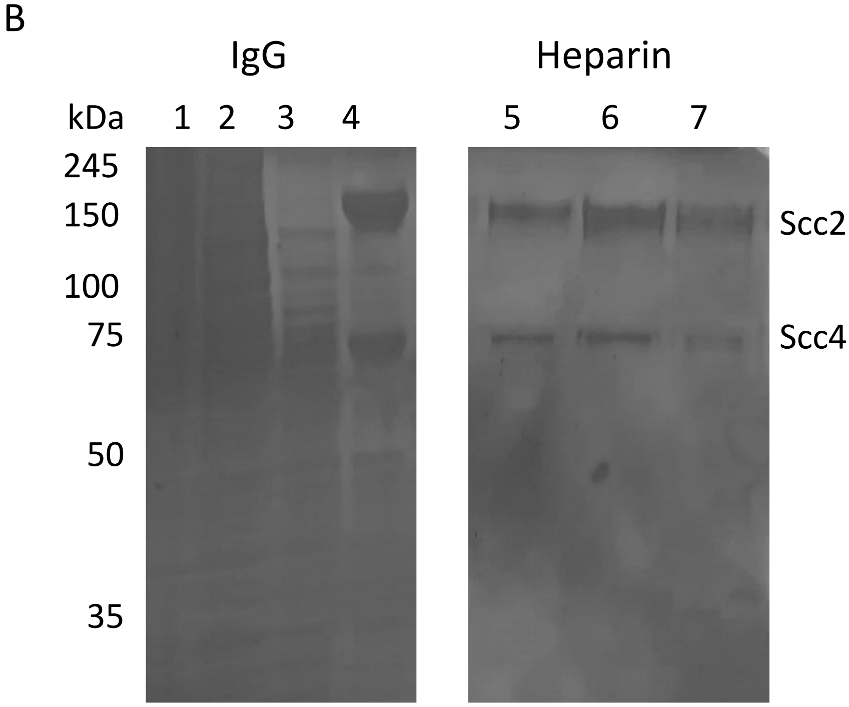
B
IgG
Heparin
kDa
1
2
3
4
5
6
7
245
150
100
75
50
35
Scc2
Scc4

Figure S5

FITC DIC Merge

No pep

ContP

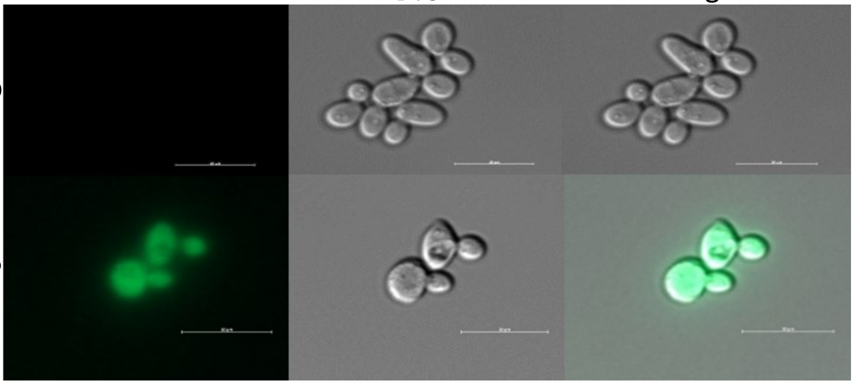

Figure S6

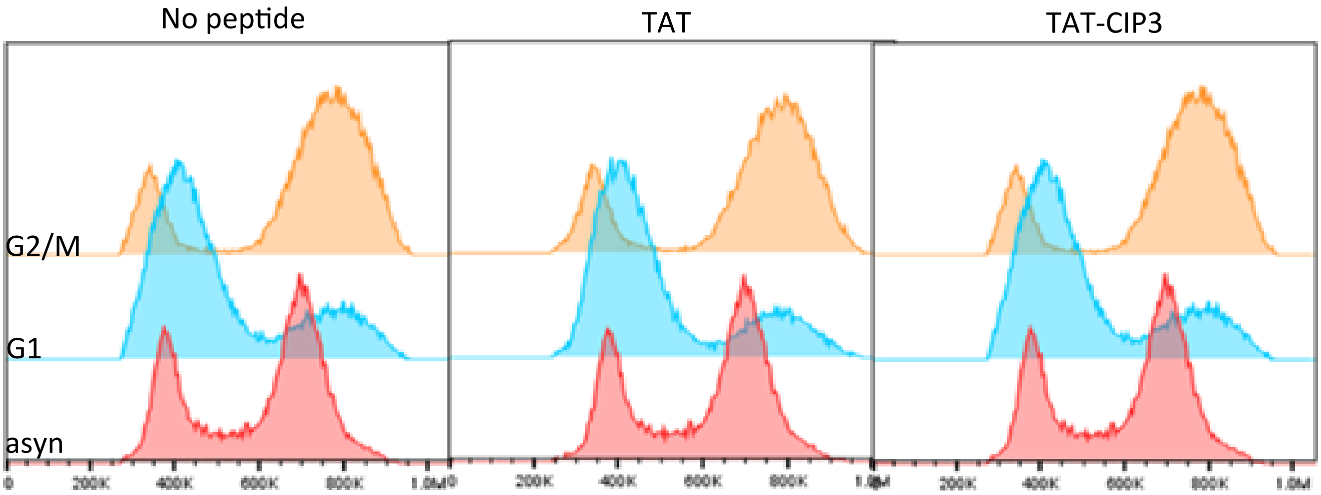

Supplementary Tables

Supplementary Table 1. Yeast strains

Strain	Description
yME-016	trp1-1 leu2-3,112 ura3-52 his3-11,15 bar1 cloNAT::lys4::LacO GFP-LacI-HIS3 pME3
yME-019	trp1-1 leu2-3,112 ura3-52 his3-11,15 bar1 cloNAT lys4::LacO GFP-LacI-HIS3 pME4
yME-031	trp1-1 leu2-3,112 ura3-52 his3-11,15 bar1 cloNAT::lys4::LacO GFP-LacI-HIS3 pME2
yIO-001	trp1-1 leu2-3,112 ura3-52 his3-11,15 bar1 GAL+
yIO-1000	trp1-1 leu2-3,112 ura3-52 his3-11,15 bar1 GAL+ URA3::pGAL-Scc4
yME-961	trp1-1 leu2-3,112 ura3-52 his3-11,15 bar1 GAL+ trp1::LacO::TRP1, his3::LacO-GFP::HIS3
yKS-008	TRP1 trp1-1 leu2-3,112 ura3-52 his3-11,15 bar1 GAL+ SMC3-V5::G418
yAM-945	trp1-1 leu2-3,112 ura3-52 his3-11,15 bar1 GAL+ SMC3-P533-GFP::SMC3
yME-047	trp1-1 leu2-3,112 ura3-52 his3-11,15 bar1 cloNAT::lys4::LacO GFP-LacI-HIS3 pAM-84
yME-035	trp1-1 leu2-3,112 ura3-52 his3-11,15 bar1 cloNAT::lys4::LacO GFP-LacI-HIS3 pAM-84, pME-012

Supplementary Table 2. Plasmid list

Plasmid	Insert	Backbone
pME2	CIP2	pRS316
pME3	CIP3	pRS316
pME4	CIP1	pRS316
pIO-014	SCC4 / YER147C	pRS316
pME-012	GFP-T2A-mCherry	pYC2A
pAM-84	GFP-T2A-CIP3	pYC2A

Supplamentry Table 3. Primer list

Primer	Direction	Description	Coordinates	Sequence
IO-12-992	Forward	ChIP/CEN3	Chr3:81023-81224	CTATTTCGAGACTGGATCCCGG
IO-12-993	Reverse		Chr3:81023-81224	GGAGACTCTTCGATAGGTGCC
IO-6-555	Forward		Chr3:111232-111376	GAAGTAATGGAAATGCCCTGATAAA
IO-6-556	Reverse		Chr3: 111232-111376	CGTTGAATGATGCCCGTAGTA
IO-3-255	Forward	ChIP/CEN4	Chr4: 4936-5018	ACACGAGCCAGAAATAGTAAC
IO-3-256	Reverse		Chr4: 4936-5018	TGATTATAAGCATGTGACCTTT
IO-3-257	Forward		Chr4: 5178-5257	CCGAGGCTTTCATAGCTTA
IO-3-258	Reverse		Chr4: 5178-5257	ACCGGAAGGAAGAATAAGAA
IO-8-681	Forward	ChIP/rDNA	Chr12:467512-467797	AGCCTACTCGAATTCGTTTCC
IO-8-682	Reverse		Chr12:467512-467797	ATAGTGAGGAACTGGGTTACC
IO-3-225	Forward	ChIP/CARC1	Chr3:99238-99418	AGC GGA TCA ATC CAC AAA GC
IO-3-226	Reverse		Chr3: 99238-99418	TGC TGT AGT CAC CTC AGC AAG
IO-3-239	Forward		Chr3:102856-102930	AAT TCC ACA GTC CCC ATA CCA C
IO-3-240	Reverse		Chr3: 102856-102930	TAC AGT GGG CGA AGT TGT GG
IO-14-1209	Forward	qPCR/pAM-84	N/A	GATTCAAAGACATGG
IO-14-1211	Reverse		N/A	CATCACCATCTAATT
